# Antiviral activity of ethanol extract of Geranii Herba and its components against influenza viruses via neuraminidase inhibition

**DOI:** 10.1038/s41598-019-48430-8

**Published:** 2019-08-20

**Authors:** Jang-Gi Choi, Young Soo Kim, Ji Hye Kim, Hwan-Suck Chung

**Affiliations:** Korea Institute of Oriental Medicine (KIOM), Korean Medicine (KM) Application Center, Daegu, 41062 Republic of Korea

**Keywords:** Drug discovery and development, Influenza virus

## Abstract

Influenza viruses are a serious threat to human health, causing numerous deaths and pandemics worldwide. To date, neuraminidase (NA) inhibitors have primarily been used to treat influenza. However, there is a growing need for novel NA inhibitors owing to the emergence of resistant viruses. Geranii Herba (*Geranium thunbergii* Siebold et Zuccarini), which is edible, has long been used in a variety of disease treatments in Asia. Although recent studies have reported its various pharmacological activities, the effect of Geranii Herba and its components on influenza viruses has not yet been reported. In this study, Geranii Herba ethanol extract (GHE) and its component geraniin showed high antiviral activity against influenza A strain as well as influenza B strain, against which oseltamivir has less efficacy than influenza A strain, by inhibiting NA activity following viral infection in Madin–Darby canine kidney cells. Thus, GHE and its components may be useful for the development of anti-influenza drugs.

## Introduction

Influenza is one of the greatest threats to human health, causing numerous deaths and sporadic pandemics worldwide^1^. Several anti-influenza drugs and seasonal influenza vaccines are available and can be generally effective. However, there have been recent situations in which the efficacy of drugs and vaccines may be limited due to drug resistance and the emergence of novel viruses with mutations^2–4^. To date, two classes of antiviral drugs have been approved to treat influenza virus infections: M2 ion channel blockers (e.g., amantadine and rimantadine)^5^ and neuraminidase inhibitors (e.g., oseltamivir and zanamivir)^6,7^. The M2 ion channel blockers block the passage of hydrogen ions to the virions in endosomes, thereby preventing the transcriptionally active viral ribonucleoprotein complex from being released into the cytoplasm^5^. However, these are seldom used in clinical medicine because many influenza viruses, including H3N2 and H5N1, are now resistant to amantadine and rimantadine^3,8,9^. Thus, NA inhibitors like oseltamivir and zanamivir are currently being prescribed for most influenza virus infections^6,10,11^. Recently, new drugs favipiravir and baloxavir marboxil, which are effective against influenza viruses by inhibiting viral RNA-dependent RNA polymerase and cap-dependent endonuclease activity, have been developed^12,13^.

NA inhibitors play an important role in preventing the spread of influenza infection by inhibiting the enzymatic function of NA, the surface glycoprotein of influenza virus, via attachment to its active site^11^. This active site of NA is a good target for the development of anti-influenza drugs as it is highly conserved among both influenza A and B viruses^10,14,15^. However, drug resistance remains a problematic issue for existing NA inhibitors. It has been reported that viruses containing the mutation H274Y (histidine to tyrosine at NA residue 274, N2 numbering) exhibit decreased NA enzymatic activity and display resistance to oseltamivir^16,17^. In addition, the E119G/D/A mutation has also been reported to cause resistance to zanamivir^18,19^. Clearly, the development of new neuraminidase inhibitors is continuously required.

Traditionally used medicinal plants are a valuable source of new drugs^20,21^. There are many reports describing the potent antiviral activity of such plants^22^, which have provided alternative therapies for the treatment of influenza^23,24^. Recently, we reported that the extracts of two traditional medicinal plants *Eupatorium fortune*^25^ and *Panax notoginseng* root^26^ showed antiviral activity against influenza virus *in vitro* and *in vivo*. Thus, the search for antiviral active agents using these natural sources provides potential for the development of new effective antiviral agents.

The dried aerial part of *Geranium thunbergii*, Geranii Herba, has been reported to effectively treat a variety of diseases such as obesity^27^, dysentery, and diarrhea^28^, and they also exhibit anti-inflammatory properties^29^. Although several studies have reported on the antiviral activities of geraniin (GN), the main component of Geranii Herba, against herpes simplex virus (HSV), human immunodeficiency virus-1 (HIV-1), dengue virus type 2 (DENV2), and human enterovirus 71 (E71), no studies have reported on the antiviral effects of Geranii Herba for the treatment of influenza virus infections^30–33^.

Based on this premise, this study aimed to evaluate the anti-influenza activity of *G*. *thunbergii* ethanol extract (GHE). We showed that controlling the process of NA inhibition plays an important role in the antiviral activity of GHE against influenza viruses. We also identified GN as the active component in GHE affecting NA inhibition. Together, these results suggest that GHE and its components are attractive candidates for the development of novel antiviral agents for the prevention and treatment of influenza viral infections.

## Results

### Effects of GHE on Madin–Darby canine kidney (MDCK) cell viability

**Figure 1:**
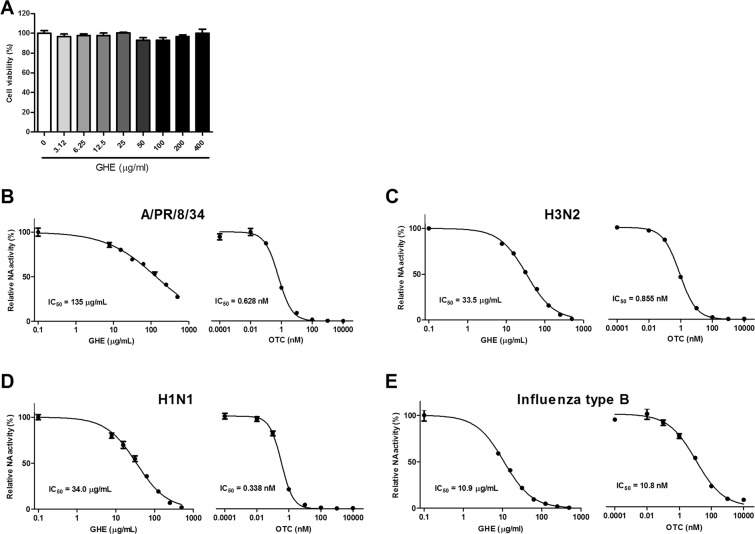
Determination of the cytotoxicity and antiviral activity of *G*. *thunbergii* ethanol extract (GHE) in MDCK cells. The viability of MDCK cells was assessed using an MTS assay after treatment with the indicated concentrations of GHE for 48 h (**A**). Measurement of the antiviral activity of GHE using neuraminidase (NA) inhibition assay. Influenza A viruses including A/PR/8/34 (**B**), H3N2 (**C**), H1N1 (**D**), and influenza type B (**E**) were added to the indicated concentrations of GHE and oseltamivir carboxylate (OTC). Fluorescence was measured using fluorescence spectrophotometry (excitation, 365 nm and emission, 415–445 nm). Bar graph (mean ± SEM) statistics were determined by three experiments’ data using one-way ANOVA with Tukey’s post-hoc test, ***P < 0.001; **P < 0.01. n.s.: not significant, compared with the (GHE untreated) samples.

GHE was tested for cytotoxicity after exposure to MDCK cells at various concentrations (0–400 μg/mL) for 48 h. Figure 1A shows the absence of a toxic effect of GHE on MDCK cell viability up to concentrations of 400 μg/mL. Thus, the cells were treated at doses lower than 400 μg/mL in subsequent experiments.

### Inhibitory effects of GHE on NA activity

NA inhibitors play an important role in preventing the spread of influenza infection via inhibition of the enzyme function of NA, the surface glycoprotein of influenza virus, by attaching to its active site^11^. Accordingly, the active site of NA is a good target for the development of anti-influenza drugs. This study investigated the potential effects of GHE on influenza virus NA activity. The NA activity of H1N1 (A/PR/8/34, and A/Korea/33/2005), H3N2 (A/Korea/32/2005), and influenza type B (B/Korea/72/2006) was significantly reduced with GHE and oseltamivir carboxylate (Fig. 1B–E). In particular, treatment with GHE (250 μg/mL) had significant effects on the NA activity of H3N2 and H1N1. We further assessed the NA activity of GHE using chemiluminescent-based neuraminidase inhibition (NI) assays. The results of this assessment confirmed that GHE inhibits NA activity in influenza A virus H3N2 similar to that demonstrated by the results of fluorescent-based NI assay (Supplementary Fig. 1A,B). Moreover, GHE exhibited 3.1–12-fold increase in NA inhibition against influenza type B strain whereas influenza B strain was much less susceptible (13–32-fold) to oseltamivir carboxylate than influenza A strain (Fig. 1B–E). The results suggest that GHE has an additional inhibitory effect on the influenza virus release stage by inhibiting the NA of A/PR/8/34, H3N2, H1N1, and type B in a dose-dependent manner.

### GHE inhibited the infection of influenza virus in MDCK cells

**Figure 2:**
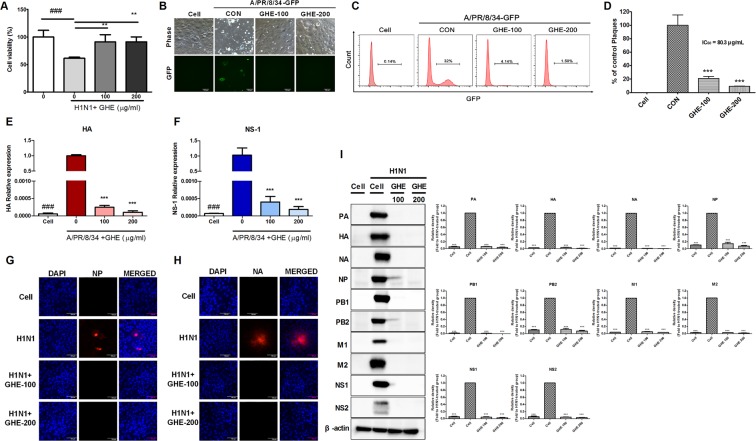
Antiviral activities of GHE on influenza A/PR/8/34, A/PR/8/34-GFP, and H1N1 viruses in MDCK cells. MDCK cells were treated with GHE (100 and 200 µg/mL) prior to influenza A virus (A/PR/8/34, A/PR/8/34-GFP, and H1N1) infection, and cells were incubated with medium alone, or 100 or 200 µg/mL GHE prior to infection with A/PR/8/34 and H1N1 (multiplicity of infection = 1). (**A**) Determination of the antiviral activity of GHE in MDCK cells. The viability of MDCK cells was assessed using an MTS assay after treatment with the indicated concentrations of GHE for 48 h after viral infection. (**B**) GFP expression levels and (**C**) reduction in viral replication using flow cytometry were assessed at 24 h after viral infection in GHE-treated MDCK cells. (**D**) Effects of GHE treatment on A/PR/8/34 infection and viral growth in plaques. The mixtures of A/PR/8/34 and GHE were incubated for 72 h after viral infection at 37 °C with 5% CO_2_. Effect of GHE components on influenza A virus mRNA synthesis and protein levels. Effect of treatment with GHE (100 or 200 µg/mL) on influenza A/PR/8/34 (multiplicity of infection = 1)-infected MDCK cells and the relative mRNA levels of influenza A/PR/8/34 HA (**E**) and NS-1 (**F**) were analyzed using quantitative real-time polymerase chain reaction and normalized to β-actin mRNA levels. Bar graph (mean ± SEM) statistics were determined by three experiments’ data using one-way ANOVA with Tukey’s post-hoc test, ***P < 0.001, compared with the CON (GHE untreated) samples. ^###^P < 0.001, compared with the cell only sample. GHE reduced the expression of influenza A virus (H1N1) proteins NP (nuclear protein) and NA in infected MDCK cells. The reduction of NP (**G**) and NA (**H**) proteins in MDCK cells were observed with fluorescence microscopy using the influenza A viral proteins NP- and NA-specific antibodies. MDCK cells were also stained with DAPI (blue), and the merged images represent NP and NA (red). Influenza H1N1 virus protein levels (NP, PA, M1, M2, PB1, PB2, HA, and NA) in MDCK cell lysates were detected using Western blotting, and β-actin was analyzed as an internal control (I). Full-length blots are presented in Supplementary Fig. 2.

To investigate if GHE inhibits influenza A virus infection in MDCK cells, we examined viral replication in GHE-treated MDCK cells (100 or 200 μg/mL) infected with H1N1 (Fig. 2). We observed that GHE-treated MDCK cells had significantly increased cell survival rate compared to the cells exposed only to H1N1 (Fig. 2A), indicating that treatment with GHE reduces viral replication in MDCK cells. Moreover, GHE-treated MDCK cells showed reduced green fluorescent protein (GFP) expression levels compared to the untreated cells with high GFP expression levels upon infection with A/PR/8/34-GFP at 24 h (Fig. 2B). Additionally, flow cytometry analysis using fluorescence detection and plaque reduction assay showed that GHE effectively inhibits viral replication in MDCK cells (Fig. 2C,D). At the highest concentration (200 μg/mL) of GHE, viral titers were reduced by 4.8 log_10_ TCID_50_/mL at 48 h post infection (Supplementary Fig. 1D). We further confirmed that GHE also inhibited viral growth in MDCK cells infected by influenza A virus at low multiplicity of infection (MOI) conditions (0.1 and 0.01) using NA-XTD influenza neuraminidase assay (Supplementary Fig. 1E,F). These results indicate that GHE-treated cells exhibited significantly reduced cell death and viral load following infection with influenza virus as compared to untreated cells.

### GHE reduced mRNA and protein levels of influenza a virus

We investigated if GHE inhibited the synthesis of influenza A HA and NS-1 mRNA. A/PR/8/34-infected MDCK cells were harvested after treatment with GHE at a concentration of 100 or 200 μg/mL, and the relative mRNA expression levels of the viral genes at 24 h were evaluated using quantitative real-time polymerase chain reaction (qRT-PCR). HA and NS-1 mRNA synthesis was completely inhibited in GHE-treated MDCK cells versus untreated MDCK cells at 24 h (Fig. 2E,F).

Furthermore, we evaluated the effect of GHE on the expression of influenza A virus proteins, such as NP (nucleoprotein) and NA (neuraminidase), using immunofluorescence analysis in GHE-treated MDCK cells at 24 h after infection with H1N1. The expression of influenza A virus proteins NP and NA was inhibited in GHE-treated MDCK cells (100 and 200 μg/mL) compared to that in the untreated cells upon infection with H1N1 at 24 h (Fig. 2G,H). In addition, the effect of GHE on the expression of influenza A virus protein NP was evaluated using immunofluorescence analysis in GHE-treated MDCK cells 24 h after infection with A/PR/8/34-GFP (MOI = 10). The expression of influenza A virus protein NP was inhibited in GHE-treated MDCK cells (100 and 200 μg/mL) whereas it was not inhibited in the untreated cells upon infection with A/PR/8/34-GFP at 24 h (Supplementary Fig. 1C). We also investigated the effect of GHE on the expression of influenza A virus proteins PA, HA, NA, NP, PB1, PB2, M1, M2, NS-1, and NS-2 using western blot analysis at 24 h after infection with H1N1. The expression of influenza A virus proteins NP and NA was inhibited in GHE-treated MDCK cells (100 and 200 μg/mL) compared to that in the untreated cells upon infection with H1N1 at 24 h (Fig. 2I). Therefore, our results indicate that GHE treatment reduces the expression of influenza A virus proteins PA, HA, NA, NP, PB1, PB2, M1, M2, NS-1, and NS-2 induced by H1N1 infection.

### Chemical composition of GHE by high performance liquid chromatography (HPLC) analysis

**Figure 3:**
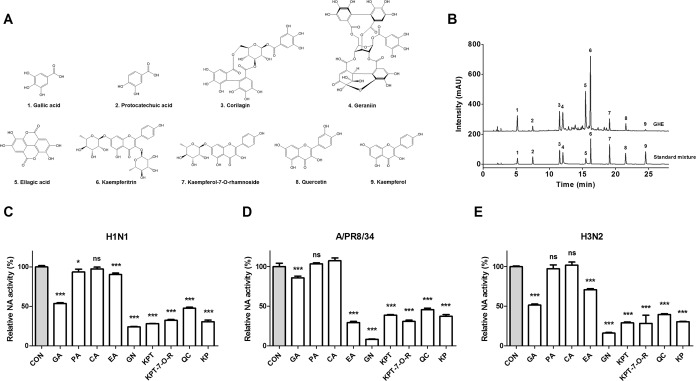
The HPLC profile of the components in GHE was monitored at 265 nm and compared with that of nine standard compounds. (**A**) Structures of the nine components of GHE: 1, Gallic acid; 2, Protocatechuic acid; 3, Corilagin; 4, GN; 5, Ellagic acid; 6, Kaempferitrin; 7, Kaempferol-7-O-rhamnoside; 8, Quercetin; 9, Kaempferol. (**B**) HPLC profile of 5 mg/mL GHE and standard compounds. Measurement of the antiviral activity of GHE components (100 μM) using neuraminidase inhibition assay. The influenza A viruses H1N1 (**C**), A/PR/8/34 (**D**), and H3N2 (**E**) were added to the indicated concentrations of GHE components. Fluorescence was measured using fluorescence spectrophotometry (excitation, 365 nm and emission, 415–445 nm). Bar graph (mean ± SEM) statistics were determined by three experiments’ data using one-way ANOVA with Tukey’s post-hoc test, ***P < 0.001; *P < 0.05, compared with the CON (GHE untreated) samples. GA, Gallic acid; PA, Protocatechuic acid, CA, Corilagin; GN, Geraniin; EA, Ellagic acid; KPT, Kaempferitrin; KP-7-O-R, Kaempferol 7-O-rhamnoside; QC, Quercetin; KP, Kaempferol.

We selected nine major and minor components of GHE through a literature survey for the profile analysis of Geranii Herba components^34–36^. We performed HPLC analysis to investigate the chemical composition of the selected GHE components by comparing the retention times with standard mixtures. Chemical analysis of GHE by HPLC revealed the presence of the following compounds: gallic acid (tR = 5.194 min; peak 1), protocatechuic acid (tR = 7.490 min; peak 2), corilagin (tR = 11.550 min; peak 3), GN (tR = 12.041 min; peak 4), ellagic acid (tR = 15.461 min; peak 5), kaempferitrin (tR = 16.251 min; peak 6), kaempferol 7-O-rhamnoside (tR = 19.101 min; peak 7), quercetin (tR = 21.502 min; peak 8), and kaempferol (tR = 24.536 min; peak 9) (Fig. 3A,B, and Table 1).

**Table 1:** The contents of phytochemicals in *G*. *thunbergii* ethanol extract.

No.	Chemical name	Retention time (min)	Contents (mg/g extract)
1	Gallic acid	5.194	13.6
2	Protocatechuic acid	7.490	3.2
3	Corilagin	11.550	25.4
4	Geraniin	12.041	39.4
5	Ellagic acid	15.461	23.9
6	Kaempferitrin	16.251	65.2
7	Kaempferol 7-O-rhamnoside	19.101	8.9
8	Quercetin	21.502	5.8
9	Kaempferol	24.536	1.2

The HPLC results also indicated that GHE contained the following components: gallic acid, 13.6 mg/g extract; protocatechuic acid, 3.2 mg/g; corilagin, 25.4 mg/g; geraniin, 39.4 mg/g; ellagic acid, 23.9 mg/g; kaempferitrin, 65.2 mg/g; kaempferol 7-O-rhamnoside, 8.9 mg/g; quercetin, 5.8 mg/g; and kaempferol, 1.2 mg/g (Table 1).

### Antiviral activity of the nine components were identified from GHE against influenza viruses

**Figure 4:**
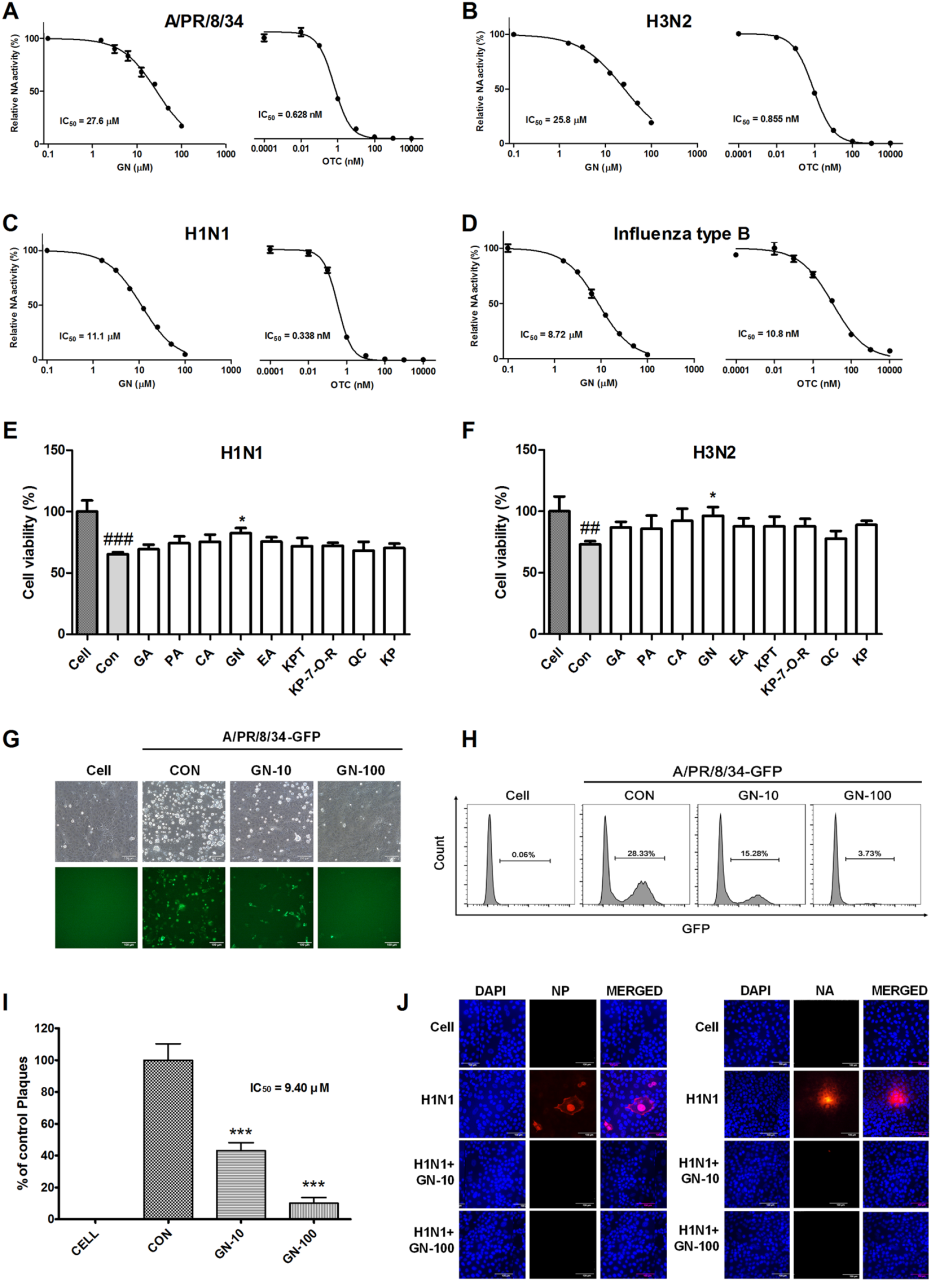
Antiviral activities of GHE components on influenza A/PR/8/34, A/PR/8/34-GFP, H1N1, influenza B viruses. Measurement of the antiviral activity of GN using neuraminidase (NA) inhibition assay. Influenza A viruses including A/PR/8/34 (**A**), H3N2 (**B**), H1N1 (**C**), and influenza type B (**D**) were added to the indicated concentrations of GN and oseltamivir carboxylate (OTC). Fluorescence was measured using fluorescence spectrophotometry (excitation, 365 nm and emission, 415–445 nm). Antiviral activities of GHE components on influenza A H1N1, H3N2, A/PR/8/34 and A/PR/8/34-GFP viruses in MDCK cells. MDCK cells were treated with constituents of GHE (100 μM) prior to influenza A virus infection, and cells were incubated with medium alone, 100 μM constituents of GHE prior to infection with A/PR/8/34 and H3N2 (multiplicity of infection = 1). The antiviral activity of the constituents of GHE was determined in MDCK cells. The viability of MDCK cells was assessed using an MTS assay after treatment with the indicated concentrations of GHE for 48 h against (**E**) H1N1 and (**F**) H3N2. (**G**) GFP expression levels and (**H**) reduction in viral replication using flow cytometry were assessed at 24 h after viral infection in geraniin (GN)-treated MDCK cells (GN: 10 and 100 μM). (**I**) Effects of GN treatment on A/PR/8/34 infection and viral growth in plaques. The mixtures of A/PR/8/34 and GN were incubated for 72 h at 37 °C with 5% CO_2_. GN reduced the expression of influenza A virus (H1N1) proteins NP and NA in infected MDCK cells to virus (**J**). The reduction of NP and NA proteins in MDCK cells were observed with fluorescence microscopy using the influenza A viral proteins NP- and NA-specific antibodies. MDCK cells were also stained with DAPI (blue), and the merged images represent NP and NA (red). Bar graph (mean ± SEM) statistics were determined by three experiments’ data using one-way ANOVA with Tukey’s post-hoc test, ***P < 0.001; *P < 0.05, compared with the CON (GHE untreated) samples. ^###^P < 0.001, compared with the cell only sample.

We further investigated the potential NA inhibition activity of these nine components identified from GHE (Fig. 3C–E). The inhibition of NA activity was confirmed using the NA-Fluor™ influenza neuraminidase assay. The nine components (at 100 μM) were added to the viruses A/PR/8/34, H1N1, and H3N2. Oseltamivir carboxylate was used as a positive control. We noted that the treatment with GN demonstrated the highest NA-inhibitory activity against all viruses (Fig. 3C–E). Furthermore, we evaluated the dose-dependent NA inhibition activity of GN and observed that GN is effective on both influenza type B and A strains, particularly H1N1 (Fig. 4A–D).

To investigate if the nine aforementioned GHE components inhibited influenza viral infection in MDCK cells, we examined viral replication in these cells treated with the nine components (at 100 μM) and infected with H1N1. We observed that GN treatment increased cell survival rate after viral infection (Fig. 4E,F), indicating that treatment with GN reduces viral replication in MDCK cells. In addition, GN-treated MDCK cells showed reduced GFP expression levels compared to the untreated cells, which displayed high GFP expression levels upon infection with PR8-GFP at 24 h (Fig. 4G). Moreover, the reduction of viral replication was confirmed using flow cytometry analysis with fluorescence detection of PR8-GFP and plaque reduction assay (Fig. 4H,I). Moreover, after 24 h of infection with A/PR/8/34-GFP, GN-treated A549 cells showed reduced GFP expression levels compared with the untreated cells with high GFP expression levels (Supplementary Fig. 3A). We investigated if GN inhibits the synthesis of influenza A M1 and NS-1 mRNA. A/PR/8/34-GFP-infected A549 cells were harvested after treatment with GN at a concentration of 10 or 100 μM, and the relative mRNA expression levels of the viral genes at 24 h were evaluated using qRT-PCR. M1 and NS-1 mRNA synthesis completely inhibited in GN-treated A549 cells versus that in untreated A549 cells at 24 h (Supplementary Fig. 3B,C). Additionally, NA-XTD™ assay using cell-based virus growth inhibition assay showed that GN effectively inhibited viral replication in A549 cells (Supplementary Fig. 3D). We also investigated the effect of GN on the expression of influenza A virus proteins NP and NA using immunofluorescence analysis 24 h after infection with H1N1. The NP and NA expression was inhibited in MDCK cells treated with GN (10 and 100 μM) compared to that in the untreated cells upon infection with H1N1 at 24 h (Fig. 4J). In addition, the effect of GN on the expression of influenza A virus protein NP was evaluated using immunofluorescence analysis in GN-treated MDCK cells at 24 h after infection with A/PR/8/34-GFP (MOI = 10). The expression of NP was inhibited in GN-treated MDCK cells (100 and 200 μg/mL) and was not inhibited in the untreated cells upon infection with A/PR/8/34-GFP at 24 h (Supplementary Fig. 3E). These results suggest that among the constituents of GHE, GN is a major effective substance for inhibiting influenza virus infection via NA inhibition.

### Protein-ligand docking simulation and pharmacophore analysis of the components in GHE

**Figure 5:**
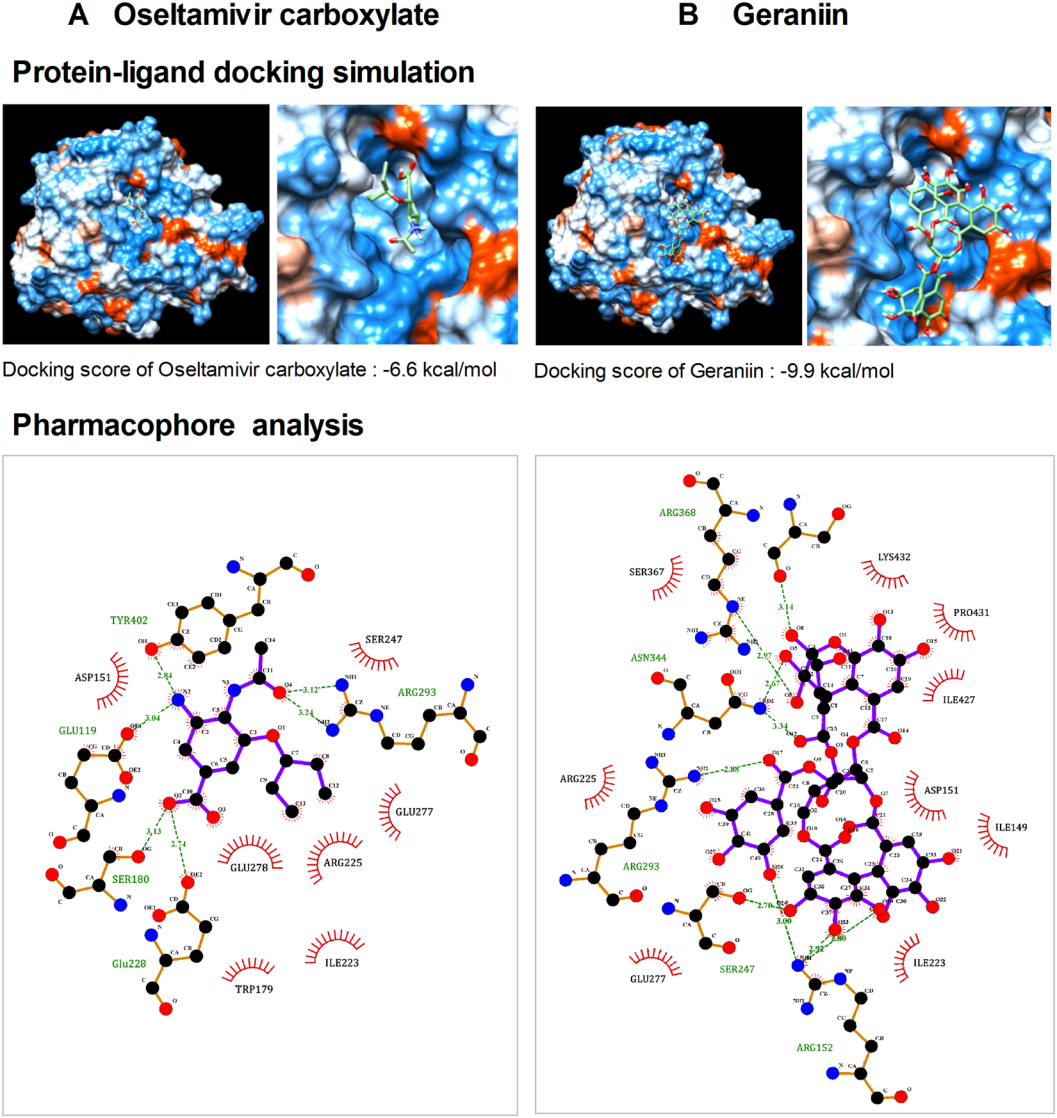
Protein docking simulation between NA and GHE components. Binding affinity of (**A**) oseltamivir carboxylate and (**B**) geraniin with NA (09H1N1, PDB ID: 3TI6) was predicted by protein docking simulation using AutoDock Vina. LigPlot+ software was applied to analyze their key hydrophobic and hydrogen bonds.

Since oseltamivir carboxylate inhibits the release of progeny virions from infected host cells by binding to NA, we investigated the binding affinity of two components in GHE to H1N1 NA (PDB code: 3TI6) by using the protein-ligand docking simulation with AutoDock Vina; these components were effective in NA inhibition as per above experiments. The predicted binding affinity between H1N1 NA and oseltamivir carboxylate was −6.6 kcal/mol (Fig. 5A). The binding affinity for GN, which is present at high concentrations in GHE, was −9.9 kcal/mol (Fig. 5B), indicating that it strongly binds to H1N1 NA corresponding to the resultant inhibition of H1N1 and A/PR/8/34 (GN). Despite the higher affinity of GN observed using docking simulation, in reality, the NA-inhibitory activity of oseltamivir is higher than that of GN, which means that more intensive studies on the relationships between activity and binding structure, such as binding direction and interactions, are required to predict the NA-inhibitory activity. Nevertheless, we further investigated the interactions between the residues of H1N1 NA and GN to assess the NA-inhibitory activity through pharmacophore analysis using LigPlot + software. Oseltamivir carboxylate is predicted to bind to H1N1 NA via six hydrogen bonds and seven hydrophobic interactions, involving the critical residues R225 and E277, which contribute to the creation of a hydrophobic pocket where the hydrophobic pentyl ether side chain of oseltamivir gets occupied by the rotation of E277 toward R225^37^. GN can form eight hydrogen bonds and nine hydrophobic interactions with the residues of H1N1 NA, including R225 and E277. The docking simulation between GHE and NA suggests that GN inhibits NA activity by binding to the hydrophobic pocket of NA with high affinity (Fig. 5B).

## Discussion

Geranii Herba (GH), belonging to the family of Geraniaceae, is a perennial plant found in Asia (Korea, China, and Japan) and has been traditionally used as anti-diarrhetic drug in East Asia^27^. GH is listed on Korean pharmacopeia and Korean Food Standards Codex. It means that GH is safe to use for additives in foods. Although there is no report on toxicity studies of GH, GH would have a low toxicity because GH allow to use food additives according to Korean Food Standards Codex. When we consider that many effective drug candidates are dropped because of their toxicities, GH would be good drug candidate. Recently, it has been studied that GH has anti-obesity effects via improvement of lipid metabolism^27^ and anti-inflammatory effects by Nrf2 activation^29^.

Despite evidence of its many medicinal uses against obesity, diarrhea, and dysentery, there have been no reported antiviral effects of GH, including therapies for the treatment of influenza virus infections.

In this study, we found that GHE inhibits the infection of influenza viruses by NA suppression and increased the survival of MDCK cells infected with influenza viruses. In addition, HA and NS-1 were not detected in qRT-PCR of A/PR/8/34-infected MDCK cells, suggesting that GHE has an antiviral mechanism for preventing the export of replicated viruses from infected cells. We further identified the constituent compounds present in GHE using HPLC and confirmed that GN exhibits antiviral effects. In addition, we investigated that the amounts of identified phytochemicals ranged from 1.2 to 65.2 mg/g in GHE extract, and kaempferitrin and GN were found to be the most abundant at 65.2 and 39.4 mg/g, respectively (Table 1).

Based on these experimental results, the NA-inhibiting properties of the nine GHE components were investigated using NA inhibition assay as well as docking simulation and interaction analysis. Among the GHE constituents, GN showed the most promising NA-inhibitory effect. GN is one of the ellagitannins which contain up to 10% of GH. GN is present in different parts of various types of plants and exhibits antiviral activity against HSV-1 and -2^30^, HIV-1^38^, HBV^39^, human enterovirus 71, and dengue virus type-2^40^. However, to date, there has been no report on the specific uses of GN in the inhibition of the NA activity of influenza viruses.

We investigated the mechanism of NA-inhibitory action of GN. We performed *in silico* modeling to investigate whether GN binds directly to the receptor NA protein of influenza virus. Pharmacophore assays were then used to identify key NA protein inhibitor interactions and common properties with the known binding compound oseltamivir carboxylate. Docking simulations performed with AutoDock Vina predicted the binding affinity of GN and oseltamivir carboxylate with the NA protein, which showed that GN is a potential NA inhibitor. This is the first report showing that GHE has anti-influenza virus activity. In addition, we showed that GN had greater anti-influenza activity than the other constituent compounds of GHE, suggesting that GN was an important active component of GHE. Interestingly, GN is known to be hydrolyzed to corilagin, gallic acid, ellagic acid, and acid after 1 h of decoction^35^. Bastian *et al*. showed that GN increase until 10 min into decoction, after that, it reduced rapidly. So, when we use a GN as an active compound in Oriental clinic, GH should be decocted for 10 min to maximize extraction of GN and minimize of its hydrolysis. Although we showed some evidences to demonstrate the antiviral effects of GHE and its compounds, further research is required to identify additional pharmacological mechanisms of GHE and to investigate GN as prophylactic and therapeutic agents against influenza viral infection through *in vivo* experiments. Additionally, further comparative analysis between GN structure and NA sequence depending on influenza virus types may provide a more interesting interpretation for the anti-influenza activity of GN. In the early 2000s, NA inhibitor (e.g. oseltamivir and zanamivir)-resistant influenza viruses emerged following mutations in E119, H274, R292, and N294 of NA^41,42^; thus, the research regarding the efficacy of natural products, including GHE and GN, against these resistant viruses will be also of interest.

In conclusion, this study was the first to demonstrate the efficacy of GHE against influenza viruses and proposes an alternative to the existing anti-influenza drugs. GHE inhibited the infection of influenza virus by NA suppression and increased the survival of MDCK cells infected with influenza virus. GHE and its components, including GN, may be useful as a therapeutic or prophylactic agent for restricting viral replication via NA inhibition. In addition, GHE and its components could be a useful starting material for the development of potent pharmaceutical drugs for selected influenza virus infections.

## Materials and Methods

### Preparation of GHE

GHE was purchased from the NIKOM (National Development Institute of Korean Medicine, Gyeongsan, South Korea). The freeze-dried extract powder was dissolved in DMSO and centrifuged at 12,000 rpm for 20 min to remove the insoluble residues, following which the supernatant was stored in desiccators at 4 °C until further use.

### Cells and viruses

MDCK and A549 human lung epithelial cells were obtained from the American Type Culture Collection and maintained in Dulbecco’s modified eagle medium (DMEM) (Lonza,Walkersville, MD, USA) containing 10% fetal bovine serum (FBS; Biotechnics Research, Lake Forest, CA, USA) and 1% penicillin and streptomycin each (Cellgro, Manassas, VA, USA) at 37 °C in a 5% CO_2_ incubator. Influenza virus strains were grown and titrated as previously described^26^. In this work, we performed antiviral study with influenza A/Puerto Rico/8/34 (A/PR/8/34) and GFP-tagged A/PR/8/34 (A/PR/8/34-GFP) viruses, which were used in the previous studies^26,43,44^. Briefly, A/PR/8/34-GFP was constructed by fusing GFP gene to the C-terminal end of nonstructural protein 1 open reading frame (NS1 ORF), containing the silent mutation at the splice acceptor, without the stop codon, followed by autoproteolytic site and the nuclear export protein (NEP). Other influenza A (H1N1, A/Korea/33/2005; H3N2, A/Korea/32/2005) and influenza B (B/Korea/72/2006) viruses were purchased from the Korea Bank for Pathogenic Viruses.

### Reagents

Oseltamivir carboxylate was purchased from AOBIOUS Inc. (Gloucester, MA, USA). Gallic and protocatechuic acids were purchased from Sigma-Aldrich (St. Louis, MO, USA). Corilagin, GN, ellagic acid, kaempferitrin, kaempferol-7-O-rhamnoside, quercetin, and kaempferol were purchased from ChemFaces (Wuhan, China), and antibodies targeting influenza proteins (PA, HA, NA, NP, PB1, PB2, M1, M2, NS-1, and NS-2) were procured from GeneTex (San Antonio, TX, USA). Anti-β-actin was purchased from Cell Signaling Technology (Cell Signaling Technology, Boston, MA, USA).

### MTS assay

Cell viability was determined by using the CellTiter 96® AQueous One Solution Cell Proliferation Assay (Promega, Madison, WI, USA), according to the manufacturer’s instructions. MDCK cells (1 × 10^4^ cells/well) were seeded into 96-well plates and GHE was added to the wells at concentrations of 0–400 μg/mL. After 48 h, MTS solutions were added to each well and the cells were incubated for additional 2 h. Subsequently, absorbance at 490 nm was recorded using a GloMax® Explorer Multimode Microplate Reader (Promega, Madison, WI, USA). The values of MTS assay were represented by the mean ± SEM of four independent experiments.

### Antiviral assay

The inhibition of viral replication was assayed as previously described^25^. Briefly, MDCK cells were cultured in 96-well plates (2 × 10^4^ cells/well) for 16 h. Then differing GHE concentrations (100 or 200 μg/mL) were added to H1N1 [multiplicity of infection (MOI) = 1] and A/PR/8/34-GFP (MOI = 1) and the mixtures were incubated at 37 °C for 1 h. MDCK cells were infected with these mixtures at 37 °C for 2 h. Afterwards, the virus was removed, and cells were washed three times with PBS, and the medium was replaced by complete DMEM. The cells were incubated for 48 h at 37 °C with 5% CO_2_. The reduction of viral cytopathic effect was determined by measuring cell viability measured using MTS assay as described above^25^. Influenza virus GFP expression was measured under a fluorescence microscope (Olympus, Tokyo, Japan) following 24 h of viral infection. In addition, antiviral activities of GHE components were evaluated at 100 μM concentration by the same method described above. The values of antiviral assay were represented by the mean ± SEM of four (GHE) and three (GHE components) independent experiments.

### Plaque reduction assay

For the plaque reduction assay^45^, we used a slightly modified version of the previously used plaque reduction assay. Briefly, MDCK cells were cultured in 12-well plates (5 × 10^5^ cells/well) for 18 h. Subsequently, A/PR/8/34 (MOI = 1) was mixed with different concentrations of GHE (100 and 200 μg/mL) and GN (10 and 100 μM), and the mixtures were incubated at 37 °C for 1 h. Then, MDCK cells were infected with these mixtures at 37 °C for 2 h. Subsequently, the virus was removed, the cells were washed three times with PBS, and the MDCK monolayer was covered with 1.5% agarose with 2X complete DMEM and TPCK trypsin at 2 mg/ml. The entire MDCK monolayer along with agarose was cultivated for 3 days at 37 °C with 5% CO_2_, fixed with 10% formalin, stained with 1% crystal violet solution, and the plaques were then counted.

### TCID_50_ assay

The TCID_50_ (tissue culture infective dose 50%) method was used to measure the titer of influenza virus in MDCK cells. Serial 10-fold dilutions of the samples were incubated with cells thrice for 48–72 h. The highest dilution of the virus, still associated with 50% cytopathic effect, was used in all three wells to estimate the amount of virus present in the original suspension according to the Reed–Muench method^46^.

### Analysis of GFP expression using flow cytometry

MDCK cells were cultured in 12-well plates (5 × 10^5^ cells/well) for 18 h. Next, A/PR/8/34 (MOI = 1) was mixed with different concentrations of GHE (100 and 200 μg/mL) and GN (10 and 100 μM), and the mixtures were incubated at 37 °C for 1 h. Then, MDCK cells were infected with these mixtures at 37 °C for 2 h. Subsequently, the virus was removed, and the cells were washed three times with PBS and the medium was replaced by complete DMEM. The cells were incubated for 24 h at 37 °C with 5% CO_2_. The reduction of viral infection effect was determined by measuring GFP expression using flow cytometry. MDCK cells were harvested and resuspended in 1 mL of PBS containing 2% FBS and fixed in suspension with 4% paraformaldehyde. The cells were washed three times with PBS and stored at 4 °C until analysis with a CytoFLEX flow cell counter (Beckman). We analyzed the data using FlowJo software.

### HPLC analysis

The components of GHE were analyzed using previously reported methods with slight modifications^47^. The analyses were performed using an Alliance e2695 (Waters Corp., Milford, MA, USA) by injecting 10 μL of standard and 5 mg/mL GHE samples into a Geminin C18 column (5 μm, 250 × 4.6 mm; Phenomenex Inc., Torrance, CA, USA) at an oven temperature of 40 °C. The mobile phase was applied at a flow rate of 1.5 mL/min with a gradient of acetonitrile (A) and distilled water (B) containing 1% acetic acid as follows: 0%–50% A (0–30 min), 50%–100% A (30–35 min), 100% A (35–35.5 min), 100%–0% A (35.5–36 min), and then 0% A (36–39 min). The samples were monitored under UV light at 265 nm. The following nine compounds were used as standards: gallic acid, protocatechuic acid, corilagin, GN, ellagic acid, kaempferitrin, kaempferol-7-O-rhamnoside, quercetin, and kaempferol.

### NI assay

The NI assay was performed using an NA-Fluor™ Influenza Neuraminidase Assay and NA‐XTD™ Influenza Neuraminidase Assay Kit (Applied Biosystems, Foster City, CA, USA) as per the manufacturer’s instructions with slight modifications^22,24^. For the NA-Fluor™ influenza neuraminidase assay, GHE was added to the assay buffer in 96-well plates at concentrations of 0–500 μg/mL for A/PR/8/34, H3N2, H1N1, and influenza type B viruses. A/PR/8/34, H1N1, H3N2, or influenza type B in assay buffer were added to the GHE-containing wells and incubated at 37 °C. Oseltamivir was considered as the positive control in the assay. After 30 min, NA-Fluor Substrate was added to each well and incubated for additional 2 h, followed by recording the fluorescence (excitation: 365 nm; emission: 415–445 nm) with a fluorescence spectrophotometer (Promega, Madison, WI., USA). Samples treated with only GHE or its components were used as negative controls. In addition, the NA activities for 100 μM of GHE components were evaluated by the same method described above. As a positive control, NA activity of oseltamivir carboxylate was measured in a range of 0 to 10,000 nM. The values of NI assay were represented by the mean ± SEM of three independent experiments.

For the NA‐XTD™ influenza neuraminidase assay, GHE was added to the assay buffer in 96-well plates at the concentrations of 0–500 μg/mL for H3N2. Subsequently, H3N2 in assay buffer were added to GHE-containing wells and incubated at 37 °C. Oseltamivir was considered as the positive control in the assay. After 20 min, NA-XTD™ substrate was added to each well and incubated for additional 30 min. Then NA-XTD™ accelerator was added to each well, followed by recording the luminescence using a luminescence plate reader (Promega, Madison, WI., USA).

### qRT-PCR

The qRT-PCR assay was conducted as previously described^25^. Briefly, total RNA was extracted from H1N1-infected MDCK cells treated or untreated with GHE (100 or 200 μg/mL) using an RNeasy Mini kit (Qiagen, Hilden, Germany). qRT-PCR was carried out using an AccuPower® 2× Greenstar qPCR Master Mix (Bioneer, Daejeon, South Korea) and a CFX96 Touch Real-Time PCR System (Bio-Rad, Hercules, CA, USA) according to the manufacturers’ instructions^25^. The primer sense and antisense sequences used were as follows: HA (5′-ttgctaaaacccggagacac-3′ and 5′-cctgacgtatttgggcact-3′); NS1 (5′-gcgatgccccattccttg-3′ and 5′-atccgctccactatctgctttc-3′) and canine β-actin (5′-tgccttgaagttggaaaacg-3′ and 5′-ctggggcctaatgttctcaca-3′). The values of qRT-PCR were represented by the mean ± SEM of three independent experiments.

### Docking simulation and interaction analysis

The components of GHE and the positive control oseltamivir carboxylate were docked onto the predefined binding pocket of the H1N1 NA crystal structure (PDB code: 3TI6) retrieved from the Protein Data Bank (www.rcsb.org) using AutoDock Vina integrated with UCSF Chimera-alpha v1.13. After docking simulation, the lowest energy scoring binding mode for each compound was selected. The hydrogen bonding and hydrophobic interactions between H1N1 NA and each compound were investigated with LigPlot + v1.4.5. Amino acid residues involved in the interactions were indicated with green (H-bonds) and red (hydrophobic interactions).

### Immunofluorescence staining

For the immunofluorescence analysis, we used a slightly modified version of the previously used immunofluorescence analysis method^26^. Briefly, MDCK cells were cultured in 4-well tissue culture slides (5 × 10^4^ cells/well) for 16 h. Subsequently, H1N1 (MOI = 1) and A/PR/8/34-GFP (MOI = 10) were mixed with different concentrations of GHE (100 and 200 μg/mL) and GN (10 and 100 μM), and the mixtures were incubated at 37 °C for 1 h. MDCK and A549 cells were infected with these mixtures at 37 °C for 2 h. Thereafter, the virus was removed, and the cells were washed three times with PBS and were cultured in a CO_2_ incubator at 37 °C for 24 h. The cells were then washed three times with cold PBS and fixed with 4% paraformaldehyde in PBS and 1% Triton X-100 for 10 min each at room temperature. After blocking, the fixed cells were incubated overnight at 4 °C with NP- and NA-specific antibodies, washed three times (5 min per wash) with TBS, and incubated with Alexa Fluor 568 goat anti-rabbit IgG antibody (1:1,000; Life Technologies, Eugene, OR, USA) and washed three times (5 min per wash) with TBS. Next, the cells were incubated with DAPI for 10 min and measured using fluorescence microscopy.

### Western blot analysis

MDCK cells were cultured in 6-well plates (1 × 10^6^ cells/well) for 18 h. Then, H1N1 was mixed with different concentrations of GHE (100 and 200 μg/mL), and the mixtures were incubated at 37 °C for 1 h. MDCK cells were infected with these mixtures at 37 °C for 2 h. Afterwards, the virus was removed, the cells were washed three times with PBS, and the medium was replaced by complete DMEM. After 24 h, the cells were harvested and subjected to western blotting using whole cell extracts^26^. The PVDF membrane was then blocked with 5% BSA in TBS-T buffer for 1 h and overnight at 4 °C with primary anti-PA, -HA, -NA, -NP, -PB1, -PB2, -M1, -M2, -NS-1, -NS-2, and -β-actin antibodies (1:1,000 dilution). Primary antibodies were washed three times (5 min per wash) with TBS-T buffer and incubated with HRP-conjugated secondary antibodies (1:5,000 dilution) at room temperature for 1 h; the relative intensities of protein bands were measured using Image J program^26^. The experiment was carried out three times independently, and similar results were obtained each time.

### Statistical analysis

Data are expressed as mean ± SEM. Differences in the mean values between the treatment and control groups were determined to be statistically significant by using one way ANOVA was performed with Tukey’s post-hoc test for multiple comparisons. Analyses were performed using GraphPad PRISM software® Version 5.02 (GraphPad, La Jolla, CA, USA). P < 0.05 was considered to denote statistical significance.

## Supplementary information

Supplementary Figures

## Data Availability

The datasets in this study are available from the corresponding author on reasonable request.
