# Supplementary material for: Antiviral activity of ethanol extract of Geranii Herba and its components against influenza viruses via neuraminidase inhibition
Source: Sci Rep. 2019 Aug 20;9:12132. doi: 10.1038/s41598-019-48430-8 (PMC6702199; doi:10.1038/s41598-019-48430-8)
Supplement: Supplementary file 1 — Supplementary Figures [file 41598_2019_48430_MOESM1_ESM.docx]

Antiviral activity of ethanol extract of Geranii Herba and its components against influenza viruses via neuraminidase inhibition.

Jang-Gi Choi ^†^, Young Soo Kim ^†^, Ji Hye Kim, Hwan-Suck Chung ^*^

Korea Institute of Oriental Medicine (KIOM), Korean Medicine (KM) Application Center, Daegu, 41062, Republic of Korea

^*^ Corresponding Author: e-mail: hschung@kiom.re.kr

^†^ Contributed equally

**Supplementary Materials**

**Preparation of GHE.** GHE was purchased from the NIKOM (National Development Institute of Korean Medicine, Gyeongsan, South Korea). The freeze-dried extract powder was dissolved in DMSO and centrifuged at 12,000 rpm for 20 min to remove the insoluble residues, following which the supernatant was stored in desiccators at 4°C until further use.

**Reagents.** Antibodies targeting influenza proteins (PA, HA, NA, NP, PB1, PB2, M1, M2, NS-1, and NS-2) were obtained from GeneTex (San Antonio, TX, USA). Anti-β-actin was purchased from Cell Signaling Technology (Cell Signaling Technology, Boston, MA, USA).

**Cell culture.** Madin–Darby canine kidney (MDCK) cells were obtained from the American Type Culture Collection (ATCC) and maintained in Dulbecco’s modified eagle medium (DMEM) (Lonza,Walkersville, MD, USA) containing 10% fetal bovine serum (FBS; Biotechnics Research, Lake Forest, CA, USA) and 1% penicillin and streptomycin (Cellgro, Manassas, VA, USA) at 37°C in a 5% CO_2_ incubator. Influenza virus strains were grown and titrated as previously described. Influenza A (H1N1, A/Puerto Rico/8/34 and A/Korea/33/2005; H3N2, A/Korea/32/2005) viruses were used.

**Western blot analysis.** MDCK cells were cultured in 6-well plates (1 × 10^6^ cells/well) for 18 h. Then, H1N1 was mixed with different concentrations of GHE (100 and 200 μg/mL), and the mixtures were incubated at 37°C for 1 h. MDCK cells were infected with these mixtures at 37°C for 2 h. Afterwards, the virus was removed, the cells were washed three times with PBS, and the medium was replaced by complete DMEM. After 24 h, the cells were harvested and Western blotting was performed using the whole cell extracts. The PVDF membrane was then blocked with 5% BSA in TBS-T buffer for 1 h, and overnight at 4°C with primary anti-PA, -HA, -NA, -NP, -PB1, -PB2, -M1, -M2, -NS-1, -NS-2, and -β-actin antibodies (1:1,000 dilution). Primary antibodies were washed three times (5 min per wash) with TBS-T buffer, and incubated with HRP-conjugated secondary antibodies (1:5,000 dilution) at room temperature for 1 h; the relative intensities of protein bands were measured using Image J program (NIH, Bethesda, MD, USA) . The experiment was carried out three times independently, and similar results were obtained each time.

**Statistical analysis.** Data are expressed as mean ± SEM. Differences in the mean values between the treatment and control groups were determined to be statistically significant by using one way ANOVA was performed with Tukey’s post-hoc test for multiple comparisons. Analyses were performed using GraphPad PRISM software® Version 5.02 (GraphPad, La Jolla, CA, USA). P < 0.05 was considered to denote statistical significance.


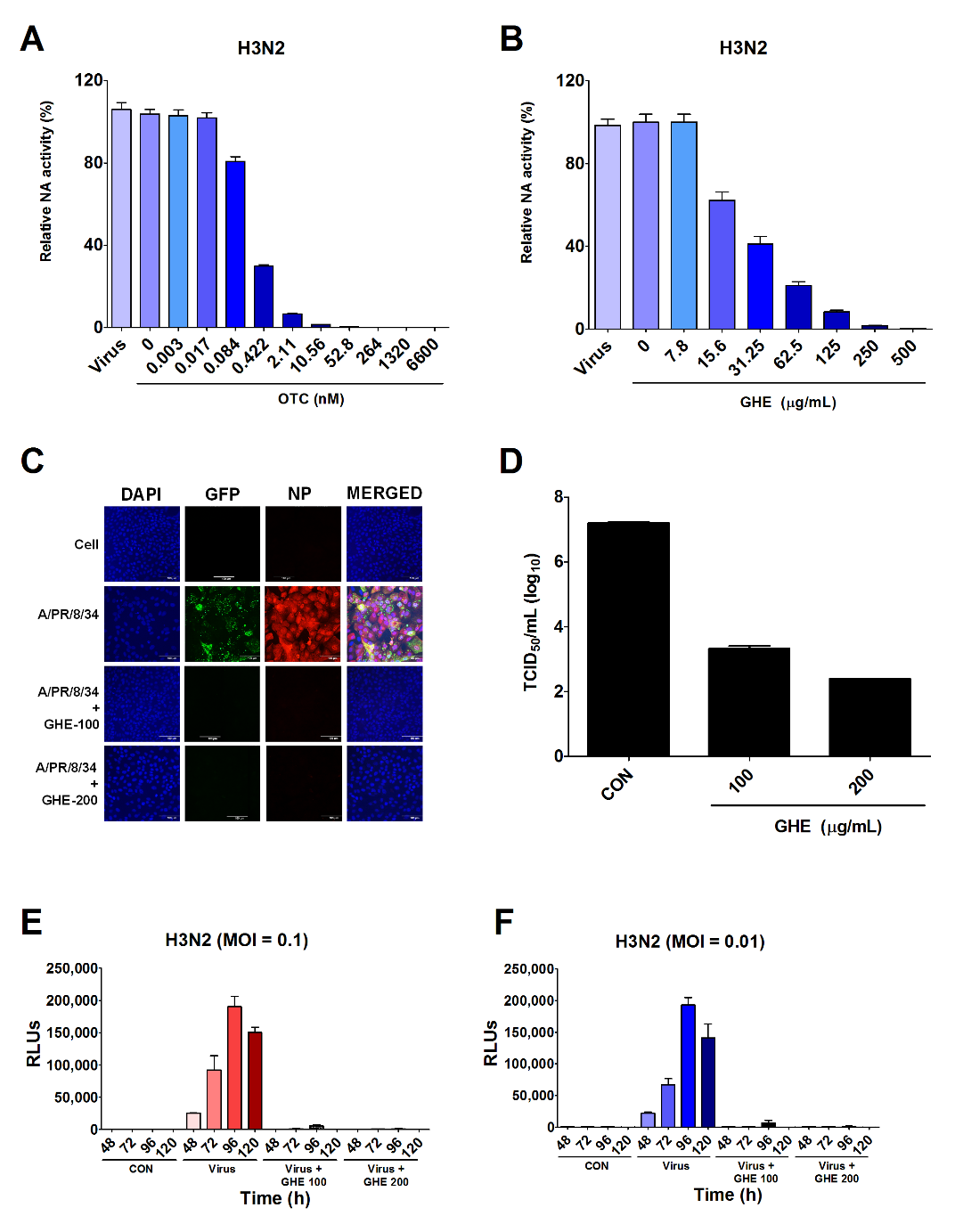


**Supplementary Figure 1.** Measurement of the antiviral activity of GHE using NA‐XTD™ Influenza Neuraminidase Assay Kit. Influenza A viruses including H3N2 were added to the indicated concentrations of oseltamivir carboxylate (A, OTC) and GHE (B). Luminescence was measured using a luminescence plate reader. Bar graph (mean ± SEM) statistics were determined using the data from three experiments by one-way ANOVA with Tukey’s post hoc test, ***P < 0.001, **P < 0.01, n.s., not significant, compared with the GHE-untreated samples. Effect of treatment with GHE (100 or 200 µg/mL) on influenza A/PR/8/34-GFP (multiplicity of infection = 10)-infected MDCK cells, the reduction of (C) NP-specific antibodies. MDCK cells were also stained with DAPI (blue), and the merged images represent NP (red). Effects of GHE treatment on A/PR/8/34 infection and viral titration. The mixtures of A/PR/8/34 and GHE were incubated for 48 h post-viral infection at 37°C with 5% CO2. (E, F) Measurement of the antiviral activity of GHE using cell-based virus growth inhibition assay (NA-XTD™ assay) after treatment with the indicated concentrations of GHE for 48, 72, 96 and 120 h post viral infection (multiplicity of infection 0.1 and 0.01).

**
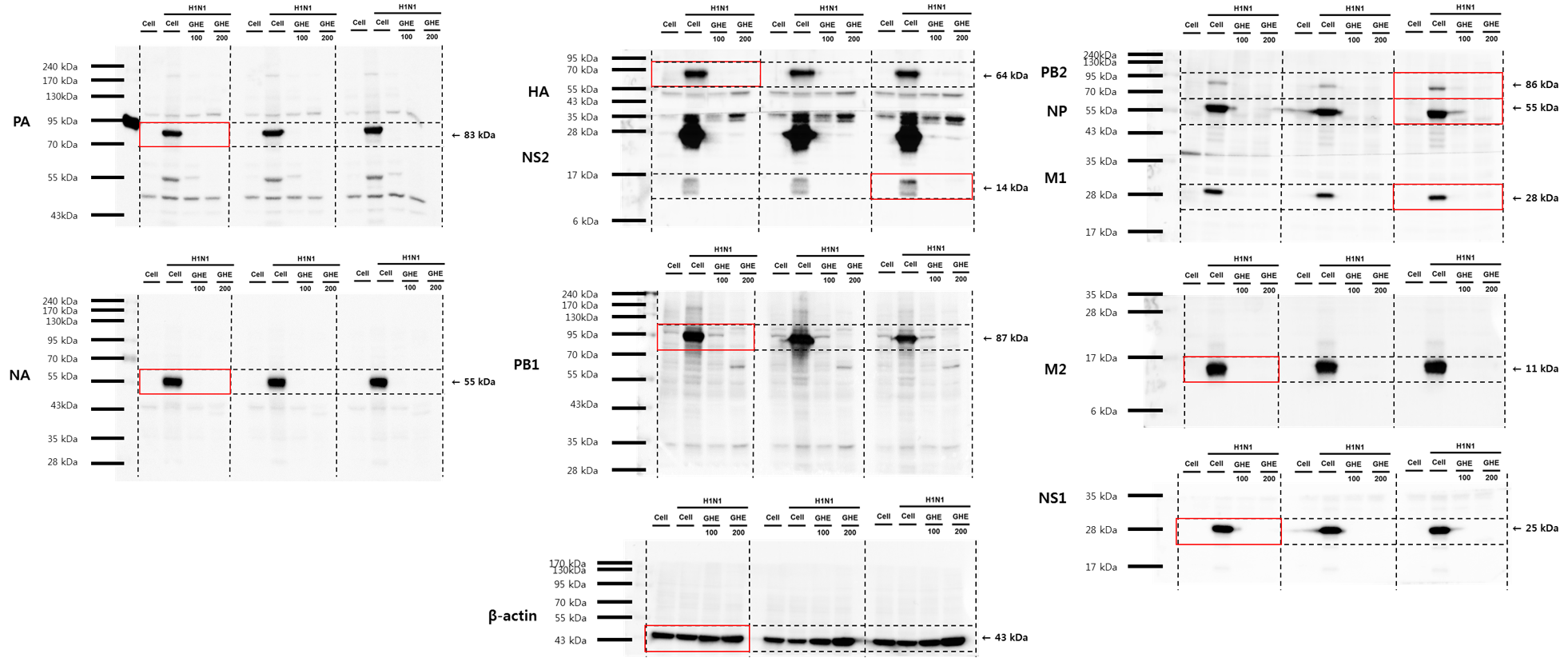
**

**Supplementary Figure 2.** Full-length images of a Western blots represented in the main figure (Fig. 2I). Red boxes delineate the images shown in figures. MDCK cells were cultured in 6-well plates (1×10^6^ cells/well) for 18 h. Then, H1N1 was mixed with different concentrations of GHE (100 and 200 μg/mL), and the mixtures were incubated at 37°C for 1 h. MDCK cells were infected with these mixtures at 37°C for 2 h. Afterwards, the virus was removed, the cells were washed three times with PBS, and the medium was replaced by complete DMEM. After 24 h, the cells were harvested and Western blotting was performed using the whole cell extracts. Influenza H1N1 virus protein levels (NP, PA, M1, M2, PB1, PB2, HA, and NA) in MDCK cell lysates were detected using Western blotting, and β-actin was analyzed as an internal control. The blots of HA and NS2 cropped from different parts of the same protein blot, the blots were separately probed using HA antibody then NS2 antibody. For PB2 and NP, the same protein samples were run in parallel to detect NP and PB2 protein using two different antibodies (αNP and αPB2). The blots of M1 cropped from different parts of the same protein blot NP and PB2, the blots were separately probed using M1 antibody. The data are representative of three independent experiments that gave similar results.


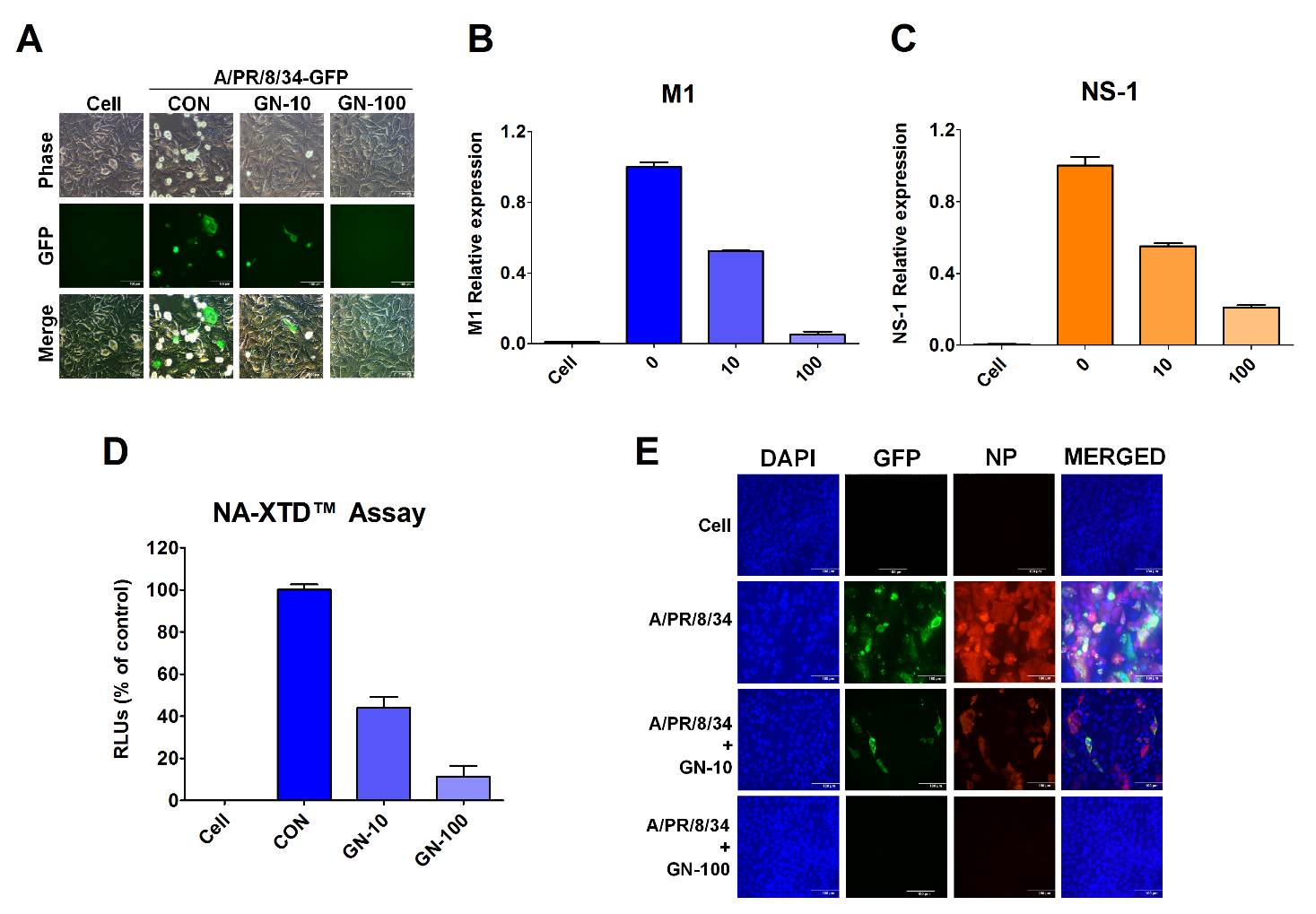


**Supplementary Figure 3.** Antiviral activities of geraniin (GN) on influenza A/PR/8/34-GFP viruses in A549 cells. A549 cells were treated with GN (10 and 100 μM) prior to influenza A virus infection, and cells were incubated with medium alone, 10 and 100 μM GN prior to infection with A/PR/8/34-GFP (multiplicity of infection = 10). (A) GFP expression levels and the relative mRNA levels of influenza A/PR/8/34 M1 (B) and NS-1 (C) were analyzed using quantitative real-time polymerase chain reaction and normalised to GADPH mRNA levels. (D) Measurement of the antiviral activity of GN using cell-based virus growth inhibition assay (NA-XTD™ Assay). GN reduced the expression of the influenza A virus (A/PR/8/34-GFP) protein NP (nuclear protein) in infected MDCK cells with A/PR/8/34-GFP (multiplicity of infection = 10). The reduction of NP (E) proteins in MDCK cells was observed with fluorescence microscopy using influenza A viral protein NP-specific antibodies. MDCK cells were also stained with DAPI (blue), and the merged images represent NP (red).
